# Deployment of novel oral polio vaccine type 2 under emergency use listing in Nigeria: the rollout experience

**DOI:** 10.11604/pamj.supp.2023.45.2.38033

**Published:** 2023-07-13

**Authors:** Adeyelu Asekun, Loveday Nkwogu, Samuel Bawa, Samuel Usman, Aboyowa Edukugho, James Ocheh, Richard Banda, Gatei wa Nganda, Peter Nsubuga, Roodly Archer, Tonia Nebechukwu, Aminu Mohammed, Faisal Shuaib, Omotayo Bolu, Usman Adamu

**Affiliations:** 1 US Centers for Disease Control and Prevention, Georgia, United States of America,; 2 World Health Organization, Abuja, Nigeria,; 3 CORE Group Polio Project, Abuja, Nigeria,; 4 African Field Epidemiology Network, Abuja, Nigeria,; 5 National Primary Health Care Development Agency, Abuja, Nigeria,; 6 Rotary International, Abuja, Nigeria,; 7 Global Public Health Solutions, Georgia, United States of America

**Keywords:** Prevalence, poliomyelitis, antibodies, immunization, risk factors, polio virus vaccine, oral, immunity, disease outbreaks, serotype, Nigeria

## Abstract

In 2011, a dedicated consortium of experts commenced work on the development of the novel oral poliovirus vaccine type 2 (nOPV2). After careful and rigorous analysis of data to enable early, targeted use of the vaccine, World Health Organization´s (WHO´s) Strategic Advisory Group of Experts on Immunization (SAGE) reviewed data from accelerated clinical development of nOPV2 and endorsed entering assessment under WHO´s Emergency Use Listing (EUL) procedure. In November 2020, nOPV2 received an interim recommendation for use under EUL to enable rapid field availability and potential wider rollout of the vaccine. In December 2020, Nigeria initiated preparation to meet all criteria for initial use of nOPV2 in the country and the documentation process to verify meeting them. The process entailed addressing the status of meeting 25 readiness criteria in nine categories for nOPV2 use in Nigeria for response efforts to ongoing cVDPV2 outbreaks. During January-February 2021, Nigeria submitted the required documentation for all required indicators for nOPV2 initial use. In February 2021, the country obtained approval from the GPEI nOPV2 Readiness Verification Team to introduce nOPV2 and in March 2021, rolled out the novel vaccine in mass vaccination campaigns for outbreak response in Bayelsa, Delta, Niger, Sokoto and Zamfara states, and one area council in the Federal Capital Territory (FCT). The lessons learned from this rollout experience in Nigeria are being applied as the country streamlines and strengthens the nOPV2 rollout process across the remaining states.

## Introduction

**Figure 1:**
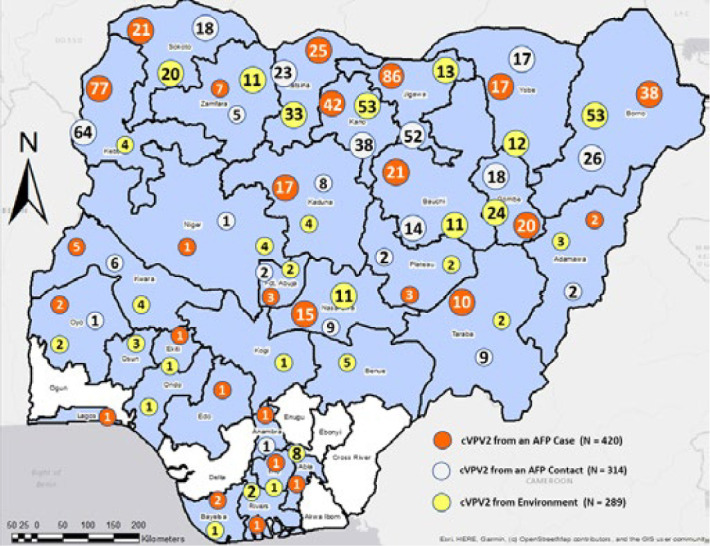
states affected by circulating vaccine derived poliovirus type 2 (cVDPV2) outbreaks and the numbers confirmed as acute flaccid paralysis cases, isolates from environmental surveillance site-positive samples, and isolates from human contacts during January 24-December 31, 2021

Great strides have been made towards polio eradication, a global initiative launched in 1988 [1]. In August 2020, the World Health Organization (WHO) Region of Africa was certified free of transmission of indigenous wild poliovirus (WPV) after Nigeria documented more than 4 years with no WPV detected [2]. Despite this outstanding achievement, the world is still at risk of a rise in polio cases if ongoing efforts are not sustained until well after the eradication goal has been achieved [3]. Outbreaks of circulating vaccine-derived poliovirus (cVDPV) of any of the three Sabin strain serotypes can emerge during prolonged person-to-person transmission in communities with low intestinal immunity and spread to cause outbreaks of paralytic disease. All vaccination using Sabin poliovirus type 2 was ceased by May 2016 to stop cVDPV type 2 (cVDPV2) outbreaks, but the number of cases and affected countries have increased since. In 2020 alone, a total of 1,021 human paralytic cases of cVDPV type 2 (cVDPV2) and 537 cVDPV2-positive environmental surveillance (sewage) samples were reported globally from 27 countries, of which 21 are countries in the WHO African Region [4]. In 2020, Nigeria reported eight cVDPV2 cases, after reporting 34 and 18 cases in 2018 and 2019, respectively. In 2021, 415 cases were reported (Figure 1) [5]. This included an increase from 11 affected states of 37 in the country (36 + the Federal Capital Territory [FCT]) in 2018 to 27 in 2021.

These outbreak numbers were driven by several compounding factors. First, historical tOPV coverage in routine immunization prior to the switch was chronically very low in several states. Second, there was an expected decline in intestinal immunity levels to type 2 poliovirus among the accumulating number of young children born after the trivalent oral polio vaccine (tOPV) switch in April 2016 [6] not otherwise exposed to oral poliovirus type 2. Additionally, following the switch, there were both insufficient routine immunization coverage with a single dose of inactivated poliovirus vaccine (which prevents paralysis for more than half vaccinees although not preventing poliovirus excretion) and low-quality outbreak response immunization campaigns with Sabin-strain monovalent oral poliovirus type 2 (mOPV2) [7]. Furthermore, the use of mOPV2 for response immunization campaigns to stop cVDPV2 outbreaks seeded new cVDPV2 emergence outbreaks in areas of low response coverage within and outside the borders of response zones [8]. Lastly, the COVID-19 pandemic halted national house-to-house polio campaigns for several months, which hindered efforts to stop polio transmission across affected countries and prevented full implementation for many months after they were restarted [4].

Given the urgent need to stop cVDPV2 outbreaks and boost population immunity against polio type 2 without leading to new emergences, a novel oral poliovirus vaccine type 2 (nOPV2) was developed and introduced in 2021 [9]. The novel vaccine has been under development since 2011 resulting from collaborations among global experts sponsored by the Bill and Melinda Gates Foundation [10,11]. The novel vaccine is a modified version of mOPV2, made to be more genetically stable for outbreak response [12]. In addition to nOPV2´s safety and efficacy in clinical trials, laboratory studies have shown the nOPV2 to be significantly less likely to revert into a form that can cause paralysis in low immunity settings compared to mOPV2 and are therefore less likely to risk seeding new emergence of cVDPV2 outbreaks [10]. Based on their review of the research, the Strategic Advisory Group of Experts on Immunization (SAGE) recommended that nOPV2 become the vaccine of choice for responding to type 2 outbreaks following an “initial use” phase. Safety and effectiveness data were required to be collected and analyzed from this initial use phase. Nigeria began preparing to meet the established criteria and submitted the necessary documentation in February 2021. The novel vaccine was authorized for use under WHO´s Emergency Use Listing (EUL) pathway in November 2020 allowing for use in outbreak response to cVDPV2 as a Public Health Emergency of International Concern [13].

Following submission of readiness documentation, Nigeria became the first country globally to be approved to use nOPV2 by the Global nOPV2 Advisory Group. This manuscript documents the lessons learned from the nOPV2 approval and rollout process in Nigeria. Documenting these lessons learned, and best practices will serve as reference material for other countries planning to introduce nOPV2 for their cVDPV2 outbreak response efforts and for the rollout of other vaccines under WHO EUL.

## Methods

**Verification process of Nigeria´s readiness for nOPV2 rollout:** in November 2020, WHO´s EUL specified the use of nOPV2 under specific guidance. To prepare for the introduction of nOPV2 into Nigeria´s cVDPV2 outbreak response efforts, a list of essential criteria and considerations were referenced and made, respectively (Table 1).

**Table 1:** essential criteria for the initial use of novel oral polio vaccine type 2 in outbreak responses; criteria and considerations for the introduction of the nOPV2 for outbreak response to circulating vaccine-derived poliovirus type 2 (cVPDV2 in a candidate country

Criteria endorsed by the Strategic Advisory Group of Experts on Immunization (SAGE)	**cVDPV2 detection**
	Capacity to acquire/distribute vaccine on time
	Capacity to respond to unanticipated findings
	Capacity for post-deployment surveillance (including safety, acute flaccid paralysis (AFP), and environmental surveillance (ES))
	A waiting period of 12 weeks after last monovalent oral poliovirus type 2 (mOPV2) use in an area
Additional consideration for nOPV2 use in outbreak responces (OBRs)	A waiting period of 6 weeks after bOPV campaigns (to minimize the risk of recombination between novel oral poliovirus vaccine type 2 (nOPV2) and bivalent oral polio vaccine (bOPV))
	Vaccine acceptance
	Access or security issues
Specifics on initial use	First use under emergency use listing (EUL): outbreak response with nOPV2 alone
	Ensure sufficient vaccine to conduct full required number of rounds with nOPV2
	IPV use for outbreak response may be considered subsequently, after the first two rounds of nOPV2

Application for EUL nOPV2 initial use required several processes implemented before, during, and after nOPV2 outbreak response [14]. Key requirements included: 1) establish a national coordinating body and technical committees to oversee the preparations for nOVP2 use in the country; 2) secure approvals for nOPV2 importation and use by relevant national and global authorities; 3) verify cold chain, logistics, and vaccine management capacity and capability and update tools to reflect the inclusion of nOPV2; 4) strengthen acute flaccid paralysis (AFP) surveillance in areas where nOPV2 is used, update case investigation forms to include nOPV2, and ensure the non-polio AFP target detection rate and AFP adequate specimen collection for the country are achieved; 5) increase environmental surveillance (ES) sewage sampling sites to include at least one functional ES site in all areas where nOPV2 is to be used and plan collection of ES samples twice per month for 6 months after nOPV2 use; 6) establish safety monitoring systems to monitor adverse events of special interest (AESIs) and adverse events following immunization (AEFIs) with nOPV2. Active surveillance for a focused list of adverse events of special interest (AESI) during the initial phase of use is an important complement to AFP and AEFI surveillance systems because it can assist with generating safety signals for complex conditions that may warrant timely further investigation to ensure public trust in the immunization program [15]. Update tools to reflect nOPV2 variables and ensure all key surveillance officers are trained; 7) develop advocacy, communications, and social mobilization strategies for key in-country stakeholders (e.g. medical practitioners, religious and community leaders) to include the Communication for Development action and crisis communication plan; 8) prepare the national laboratories for processing specimens after nOPV2 use. This entails updating the isolation algorithms and training on the intratypic differentiation (ITD) testing kits for both AFP and ES, and modifying reporting mechanisms. Relevant laboratories are prepared to promptly ship isolate specimens to the US Centers for Disease Control and Prevention (CDC) for complete genome sequencing; 9) update supplementary immunization activity (SIA) guidelines to include nOPV2 (including microplanning tools and the SIA preparedness dashboard). Develop a nOPV2 SIA training plan and materials.

**Roll-out of nOPV2 in Nigeria:** in January 2021, the Incident Manager of the National Polio Emergency Operations Center (NEOC) identified individuals from across the Global Polio Eradication Initiative (GPEI) partnership and other in-country partners to serve in newly established technical committees and provide verifiable documentation on the 25 readiness indicators for nOPV2 rollout in the country [14]. Table 2 captures these readiness indicators and their corresponding thematic areas. The team engaged in regular meetings, several consultations and reviewing sessions of Nigeria´s public health documents relevant to the documentation process. Identified leads engaged stakeholders that cut across all levels of the government, the education sector, public and private medical practitioners, and regulatory bodies. Within one month of commencing the documentation process for the readiness indicators, the assigned in-country team collated the documentation that all 25 readiness indicators were achieved, and submitted the required documents to the Global nOPV2 Advisory Group for their review and approval. The process of working to meet the criteria, collating the required information and submitting it to the Global nOPV2 Advisory Group took the national coordinating body 6 weeks. Three weeks later, nOPV2 responses were rolled out in five states and one area council in FCT.

**Table 2:** Nigeria’s readiness indicators and thematic areas - February 12, 2021

Thematic area	Indicators
Coordination	A national coordinating mechanism/body has been created and technical committees have been established to oversee preparations for nOPV2 across the following critical areas: 1) cold chain, logistics, and vaccine management; 2) safety/causality; 3) advocacy, communications, and social mobilization; 4) surveillance; and 5) laboratory
Approvals	An official decision to implement nOPV2 for outbreak response is confirmed and documented by national immunization partners
	Approval for the importation of nOPV2 has been secured from the national agency for food and drug administration and control and documented for reference
	Approval for the use of nOPV2 has been secured from the national agency for food and drug administration and Control and documented for reference
Cold chain/vaccine management	Logistics processes, vaccine management protocols, and other relevant tools for outbreak response have been adapted to reflect the characteristics of nOPV2 (i.e., containment, reverse logistics requirements, and 50-dose vial presentation)
	A cold-chain inventory assessment has been conducted or updated; freeze capacity and pre-qualified vaccine carrier availability are well documented
Acute flaccid paralysis surveillance	A plan has been developed to carry out active case searched in all priority sites in each geographic area where nOPV2 was used, one month following nOPV2 use in that area
	A plan has been developed to collect vaccination coverage data from age-matched, randomly selected community members around AFP VPD cases
	An AFP Case Investigation Form has been adapted to record routine and SIA oral poliovirus doses
	A desk surveillance review has been completed, and a plan has been developed to address identified weaknesses relevant to nOPV2 use
	A plan has been developed for systematic contact sampling of all AFP cases for 6 months after an nOPV2 outbreak response
	The country achieves a non-polio AFP rate ≥2 at the national level and in at least 80% of all districts with more than 100,000 children aged under 15 years
	The country meets stool adequacy of ≥80% at the national level and in at least 80% of all districts reporting AFP cases
Environmental surveillance	The country has at least one functional ES site in areas where nOPV2 will be used
	A plan has been developed to collect ES samples twice per month for 6 months after nOPV2 use
Safety monitoring	An active AESI safety monitoring protocol has been developed, and materials and resources at all levels are available for AEFI surveillance and AESI active case search
	All disease surveillance officers have been trained on AEFI surveillance and AESI active case search
	The causality assessment committee has been trained to conduct AEFI/AESI causality assessment and has been oriented on nOPV2 AESIs case-definitions
	The nOPV2 vaccine-related event response plan has been adapted to the country context, with stakeholder roles/responsibilities outlined and relevant training conducted
	The plan for the implementation of active safety surveillance in the local context has been finalized and ethical approvals secured if needed in conjunction with the CDC
Advocacy, communication, and social mobilization	Advocacy strategy for key in-country stakeholders (e.g., medical practitioners, religious and community leaders) has been finalized
	The UNICEF’s Communication for Development action plan has been developed. Key components include: nOPV2 communications with messaging adapted to the local context; key actors, including front-line workers have been trained; all stakeholders have been mapped and sensitized; concrete plans for digital platforms have been developed; all necessary messaging, tools, and products have been developed
	A crisis communication plan has been developed, and the plan addresses the needs identified in the nOPV2 VRE response plan for AEFI and possible public controversy (including tailored content to respond to misinformation on social media)
Laboratory	A plan has been developed to prepare the national lab for nOPV2 use, including updating the isolation algorithms and stocking/ training on the ITD testing kits for both AFP and ES along with modifications to the reporting mechanism
	Relevant laboratories are prepared to ship samples to CDC or National Institute for Biological Standards and control for complete genome sequencing for post-response monitoring
Campaign operations	SIA guidelines have been updated to include nOPV2 (including microplanning tools and the SIA preparedness dashboard)
	An nOPV2 SIA training plan and materials are developed

nOPV2: novel oral poliovirus vaccine type 2; AFP VPD: acute flaccid paralysis vaccine preventable disease; ES: environmental surveillance; AESI: adverse events of special interest; AEFI: adverse events following immunization; CDC: centers for disease control and prevention; UNICEF: united nations international children's emergency fund; VRE: vaccine-related event; ITD: intratypic differentiation; SIA: supplementary immunization activity

**Figure 2:**
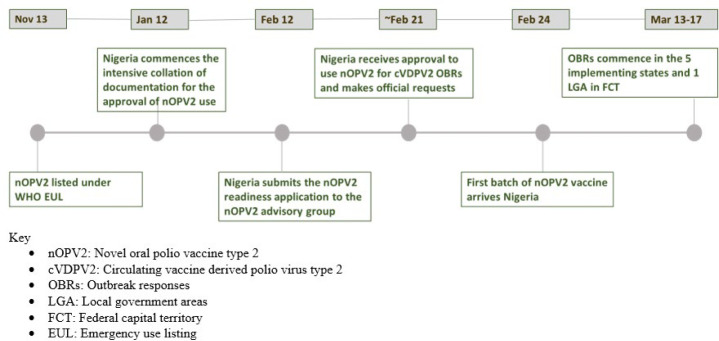
map of Nigeria indicating the five states and an area council in the Federal Capital Territory (FCT) that introduced novel oral poliovirus vaccine type 2 (nOPV2) in March 2021

**nOPV2 vaccination:** in March 2021, Nigeria rolled-out nOPV2 in cVDPV2 outbreak responses in Bayelsa, Delta, Sokoto, Zamfara, Niger states, and one Area Council in the Federal Capital Territory (FCT). Bayelsa and Delta states are located in the core of the Niger Delta region, in one of six geo-political zones designated South-South. Niger and FCT are located in the country’s North-Central zone, and Sokoto and Zamfara, in the country’s Northwestern zone (Figure 2). Lessons learned from this introduction were applied to the nOPV2 rollout in 23 additional states across the country [5]. Lot quality assurance sampling (LQAS) surveys were used as a proxy to assess the quality of the outbreak response assessment (OBRA) using nOPV2.

## Results

**Preparation for nOPV2 use:** in February 2021, the national coordinating body submitted the required documentation on the 25 readiness indicators for nOPV2 introduction, supported by national immunization partners. Additionally, the relevant authorizing agencies in the country provided the required approvals for the importation and use of the nOPV2 vaccine. Members of the polio NEOC Surveillance Working Group (SWG) worked with the technical committees to update data systems, national registers, logistics, and vaccine management protocols to implement the processes needed for use of nOPV2 with its unique characteristics. Safety monitoring systems were strengthened in all states implementing nOPV2 to monitor AESIs and AEFIs following the use of nOPV2. AESI surveillance after nOPV2 use includes AESI identification, notification, investigation, reporting, analysis and causality assessment [15]. Following the establishment of these structures and systems, a nationwide cold-chain inventory assessment was conducted to assess the impact of nOPV2 use on Nigeria´s cold chain capacity. The SWG coordinated national planning meetings to develop guidelines and training material for surveillance officers at all levels. The active vaccine safety surveillance plan was finalized, and the nOPV2 vaccine-related event (VRE) response plan adapted to the country-specific context. The members of the SWG adapted the GPEI guide for nOPV2 active surveillance to Nigeria´s context, developed training materials and updated data collection tools. The SWG then commenced training of three categories of personnel: 1) members of the National Expert Committee on AESI and causality assessment; 2) surveillance officers at the subnational level on AESI active surveillance and the collection of nOPV2-specific variables in the data tools; and 3) clinicians at the subnational level on AESI active surveillance specific to case ascertainment and data abstraction.

Surveillance officers were to conduct active surveillance for nOPV2 AESI at high-patient-load, high-priority health facility sites from the first day of vaccine use to 6 weeks following the last dose administered in the participating LGAs. The LGA surveillance officers were also to conduct weekly active surveillance visits to all other designated surveillance focal sites and review registers to identify potentially unreported, suspected cases of AEFIs and AESIs. One ad hoc ES site was established in states implementing nOPV2 responses that had no previous ES, and the frequency of ES sample collection at all collection sites was increased from once to twice per month for 6 months. A robust advocacy, communications, and social mobilization strategy and plan were developed to include nOPV2 messaging for dissemination across different platforms to the different targets of critical stakeholders. Additionally, a crisis communication plan was developed to address the response plan for AEFIs and misinformation about the vaccine. The NEOC ensured that a laboratory plan was developed to prepare the national laboratory for nOPV2 use and testing for samples for AFP cases and the potential of increased samples collected from existing and newly established ES sites. All virus isolation procedures and intra-typic differentiation in the Ibadan and Borno laboratories were followed, and isolate samples sent to the polio laboratory at United States Centers for Disease Control and Prevention for genomic sequence analysis.

**Figure 3:**
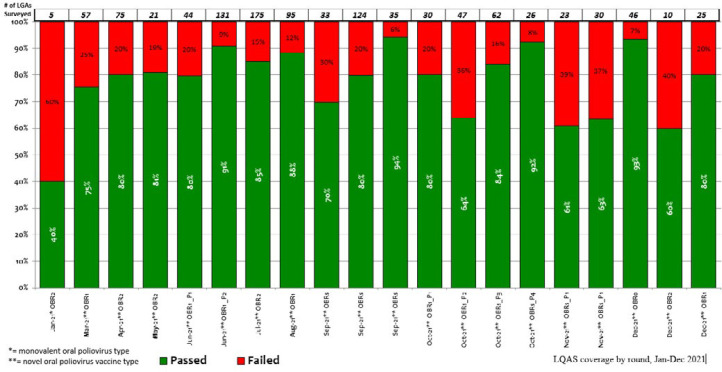
timeline for approval of novel oral poliovirus vaccine type 2 (nOPV2) Use for response supplementary immunization activities to circulating vaccine-derived poliovirus type 2 outbreaks in Nigeria, November 2020-March 2021

**Figure 4:**
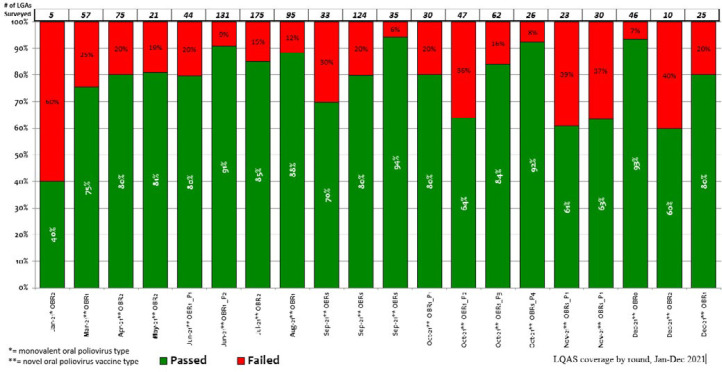
lot quality assurance sampling (LQAS) results of novel oral poliovirus vaccine type 2 (nOPV2) campaigns conducted in Nigeria from January to December 2021

**nOPV2 vaccination:** after the GPEI Outbreak Preparedness and Response Task Team received and reviewed all documentation and verified that all initial use criteria for nOPV2 use were met, it allowed release and expedited shipment of the novel vaccine by the UNICEF Supply Division. Figure 3 depicts the timeline and list of activities that occurred between November 2020 and March 2021. On February 24, the country received 12,067,500 doses of nOPV2 vaccines for the outbreak response beginning March 13 in the pilot states. In March, an additional 12,065,000 nOPV2 doses were delivered to Nigeria for the second round of the outbreak response that began April 4. During the initial nOPV2 outbreak response SIAs in five states and FCT, approximately 7.8 million children were vaccinated with nOPV2 by tally sheet records. During March-December 2021, nOPV2 vaccine was administered in cVDPV2 outbreak response SIAs across 36 states and the FCT, to over 143.6 million children (Table 3). The LQAS surveys revealed that the quality of the rounds was overall suboptimal, with variation in the number of implementing LGAs that passed the survey 90% threshold (Figure 4).

**Table 3:** novel oral polio vaccine type 2 use in response supplementary immunization activities for cVDPV2s outbreaks by month and state, Nigeria during March‒December 2021

Outbreak response month	States	No. of States/No. of LGAs	No. of children reportedly vaccinated (million)
March 2021	Bayelsa, Delta, Niger, Sokoto, Zamfara, FCT (1 area council, equivalent to a state LGA)	6/91	7.8
April 2021	Kebbi, Niger, Sokoto, Zamfara, and FCT (1 area council)	5/84	6.2
April 2021	Bayelsa and Delta	2/28	10.1
May 2021	Delta (5 LGAs) and Kebbi	2/26	2.0
June 2021	Abia, Akwa Ibom, Imo and Rivers	4/98	5.2
June 2021	Bauchi, Borno, Gombe, Jigawa, Kano, Katsina and Yobe	7/180	14.8
July 2021	Abia, Akwa Ibom, Bauchi, Borno, Gombe, Imo, Jigawa, Rivers, Kano, Katsina and Yobe	11/278	21.2
August 2021	Adamawa, Benue, FCT (5 area councils), Kaduna, Nasarawa, Plateau, Taraba and Zamfara	8/133	11.7
September 2021	Oyo and Sokoto	2/56	3.3
September 2021	Benue, FCT (5 area councils), Kaduna, Kebbi, Kwara, Nasarawa, Ogun, Osun, Plateau (14 LGAs), Taraba	10/181	13.9
September 2021	Adamawa, Lagos, Plateau (3 LGAs)	3/44	6.5
October 2021	Cross River, Edo, Kogi	3/57	2.6
October 2021	Anambra, Ebonyi, Enugu	3/51	3.4
October 2021	Sokoto	1/23	1.5
October 2021	Oyo	1/33	2.2
October 2021	Kebbi, Kwara, Ogun, Osun	4/87	5.4
October 2021	Kogi, Lagos	2/41	6.3
November 2021	Zamfara	1/14	4.9
November 2021	Edo	1/18	0.60
November 2021	Anambra	1/21	2.1
November 2021	Ebonyi, Enugu	2/30	4.6
December 2021	Bauchi (5 LGAs), Cross River, Gombe (3 LGAs), Katsina (6 LGAs)	4/32	2.1
December 2021	Jigawa (8 LGAs), Yobe (2 LGAs)	2/10	0.85
December 2021	Borno (10 LGAs), Kano (12 LGAs), Ondo	3/41	3.4
December 2021	Ekiti	1/16	0.5
December 2021	BayelsaOndo	1/8	0.48

Abbreviation - LGA: local government area; FCT: federal capital territory; cVDPV: circulating vaccine-derived poliovirus

## Discussion

The Nigeria learned several lessons in the rollout of the nOPV2 for cVPDV2 outbreak response. First, coordination is key in ensuring all requirements for nOPV2 introduction are met. WHO, UNICEF, and CDC were amongst the many in-country global immunization partners that supported the Nigeria NEOC coordinate meeting the nOPV2 criteria and the documentation process. To help facilitate engagement with the GPEI and overall coordination throughout the readiness process, countries preparing for nOPV2 use are encouraged to designate a national nOPV2 focal point [14]. We found that establishing a team focused specifically on preparing the documentation required for verification of meeting 25 criteria indicators, with overall guidance from the Incident Manager, was critical for timely criteria implementation, document collation, submission, and approval for nOPV2 release and rollout. The coordination and collaboration extended to the Federal Ministry of Health and the National Agency for Food and Drug Administration and Control, the authorizing agencies that provided the required approval for the importation and use of the nOPV2 vaccine for cVDPV2 outbreak responses. Different technical groups modified national protocols and tools to reflect the nOPV2 administration and handling, conducted a nationwide cold-chain inventory assessment and updated guidelines, training materials and data collection tools to reflect nOPV2 cVDPV2 outbreak response efforts under EUL criteria. These important steps were followed by training members of the National Expert Committee on AESI and causality assessment, and training surveillance officers and clinicians at the subnational level on AESI active surveillance/case ascertainment and data submission. This collection of steps serves as an example for the effective rollout of a new vaccine that also allowed refreshing health workers´ skills and knowledge in key aspects of immunization [16].

As new vaccines are developed, licensed, and made available for use, surveillance and monitoring are critical to addressing their impact on communities and populations and their safety [17]. To align with this, surveillance officers were trained, and existing surveillance structures were fortified to monitor and quickly detect any AEFIs following the use of nOPV2 including AFP cases. Following the training of members of the Expert Committee, they were able to conduct causality assessments to determine if there was any possible or probable causal association between any AEFI/AESI and the receipt of nOPV2. All these processes for evaluating vaccine safety during the rollout of nOPV2 can continue to improve overall monitoring of vaccine safety and pharmacovigilance. Social mobilization for immunization should be guided by a clear, data-driven strategy that is centrally positioned within the broader program [18].

There were several limitations with the nOPV2 rollout in Nigeria that also provide some lessons. First, the COVID-19 pandemic and mitigation activities put a strain on the health system and the Nigeria polio eradication program. Second, COVID-19 vaccine rollout and other state-level disease control activities coincided with cVDPV2 outbreak responses. While the polio NEOC monitored readiness to implement the outbreak response campaigns, states had to work with limited financial and workforce resources on the different activities at the state level. As a result, pre-planning activities required for high-quality campaigns were not optimal, adversely affecting the implementation of the outbreak response efforts. Third, vaccination teams were unable to adequately reach some eligible children aged <5 years in security-compromised areas (due to highway banditry or insurgent-held areas) and when they were successful, assessing SIA quality remained a challenge. Strategies such as the use of GPS tracking of vaccination teams are being used to determine which settlements were not visited and proffer strategies to address access to these areas. The tracking of vaccination teams provided significant feedback during campaigns against poliovirus in Nigeria and enabled supervisors to evaluate the teams’ performance [19]. Fourth, the limited global supply of nOPV2, made by a single manufacturer, delayed some vaccine shipments to Nigeria for the expanded rollout to all affected states [19,20]. Fifth, the documentation process required coordination across different federal agencies. With competing activities and agency-specific priorities, fostering collaboration and prioritization across the respective agencies that were stakeholders in the nOPV2 introduction required time and resulted in slight delays for some required approvals. Sixth, the cold chain logistic assessment and establishment of the safety monitoring system in states rolling out nOPV2 required funds that states did not have readily available; the federal government and partners had to support the states with this funding.

## Conclusion

Nigeria has successfully demonstrated processes for the introduction and rollout of nOPV2 for use in cVDPV2 outbreak response, albeit with some limitations in SIA quality. It is expected that continued use of nOPV2 in cVDPV2 outbreak responses will contribute to halting the spread of cVDPV2s in Nigeria if SIAs reach at-risk children with higher effectiveness than some prior SIAs [21]. For countries adopting nOPV2 for outbreak response activities, it will be important to develop strong nOPV2 readiness and safety plans that engage stakeholders at every administrative level. A team approach to planning represents a high investment in personnel time but helped to fully integrate a new vaccine into an effective outbreak response. Ensuring a coordinated roll-out of nOPV2 and improved SIA quality could substantially accelerate the global eradication goal to prevent further spread of outbreaks and stop cVDPV transmission.

**Disclaimer:** the findings and conclusions in this report are those of the authors and do not necessarily represent the official position of the U.S. Centers for Disease Control and Prevention.
